# Magnetic Resonance Imaging of Brain in Evaluation of Floppy Children: A Case Series

**Published:** 2015

**Authors:** Shantiranjan SANYAL, Sharmila DURAISAMY, Umesh Chandra GARGA

**Affiliations:** 1 Senior Resident, Department of Radiology, Dr. RML Hospital, Guru Govind Singh Indraprastha University, New Delhi, India; 2 Senior Resident, Department Rf Radiology, Dr. RML Hospital, Guru Govind singh Indraprastha, University, New Delhi, India; 3 Department of Radiology, Dr. RML Hospital, Guru Govind singh Indraprastha, University, New Delhi, India

**Keywords:** Central hypotonia, Floppy child, MRI

## Abstract

**Objective**

Hypotonia is a common clinical entity well recognized in pediatric age group, which demands experienced clinical assessment and an extensive array of investigations to establish the underlying disease process. Neuroimaging comes as great help in diagnosing the disease process in rare cases of central hypotonia due to structural malformations of brain and metabolic disorders and should always be included as an important investigation in the assessment of a floppy child. In this article, we discuss the MRI features of eight cases of central and two cases of combined hypotonia and the importance of neuroimaging in understanding the underlying disease in a hypotonic child.

## Introduction

Hypotonia is a constant clinical sign associated with a wide variety of cerebral as well as systemic diseases in newborns and older infants. Causes can be common such as hypoglycemia and hypoxic ischemic injuries that do not pose many difficulties in diagnosis and could be rare Inborn Errors of Metabolism; which are difficult to diagnose especially in resource limited Nations. A thorough clinical examination by an experienced pediatrician is usually the first step necessary to determine the anatomical origin of hypotonia i.e., whether in the central or peripheral nervous system (1). Careful physical examination and a detailed history help to tailor the investigations and decide which patients need to be referred for neuroimaging. Among a wide list of causes in both groups, establishing the underlying cause often becomes a diagnostic challenge to the clinicians. Imaging is very useful such situations. Central hypotonia accounts for 60-80% of cases of hypotonia in infants (1, 2). The most common cause for central hypotonia in newborns is hypoxic ischemic encephalopathy. Brain malformations and inborn errors of metabolism account for 13% and 3% respectively among the prevalence of the causes of hypotonia (2). Clinical signs of global developmental delay, seizures with preserved tendon reflexes and a normal creatine kinase levels point towards a central nervous system problem with the need for neuroimaging (2). Magnetic Resonance Imaging (MRI) plays a great role not only to establish the actual cause but also helps in pointing out associated anomalies, rule out other differentials and predicting the prognosis which can be explained to caregivers besides Magnetic Resonance spectroscopy (MRS) can reveal the inborn errors of metabolism. We have studied the MRI brain imaging of children presenting with central hypotonia in this article and discussed the role of MRI as a problem-solving tool in evaluating floppy children.

## Materials and Methods

In this article, MRI brain imaging details have been discussed retrospectively in eight patients of central and two cases of combined hypotonia referred to the Department of Radiology of Dr. Rml Hospital, a Tertiary Care Institute for neuroimaging in northern India. We have excluded the more common systemic or acquired causes like sepsis or hypoxic ischemic encephalopathy and those cases where MRI was essentially normal. MRI was performed on a 1.5 Tesla Siemens Magnetom Symphony scanner with 4 mm thick slices and 2 mm interslice gap. All standard sequences were included including post contrast sequences and MR Spectroscopy in special situations as and when required.

## Results

**MRI findings in structural anomalies**

**Fig 1:**
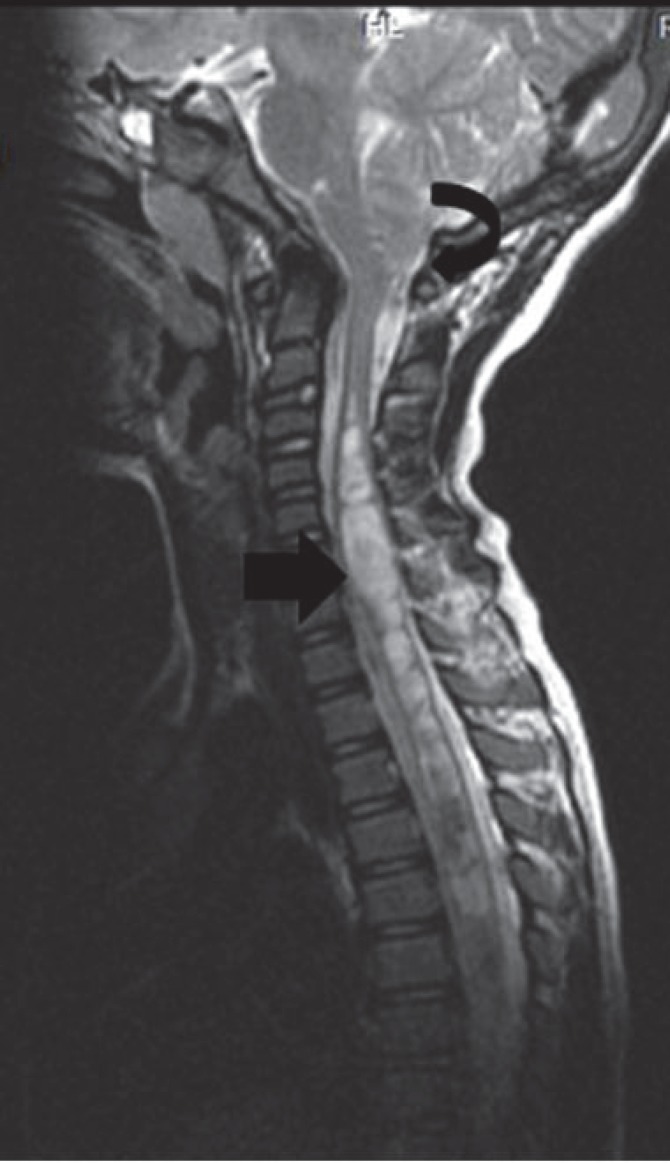
Saggital T2 W image shows herniation of cerebellar tonsil(curved arrow in a) into the cervical canal (10mm) with syringomyelia (straight arrow)involving whole of spinal cord in a case of Chiari I with holochord syrinx in an 8 year child

**Fig 2:**
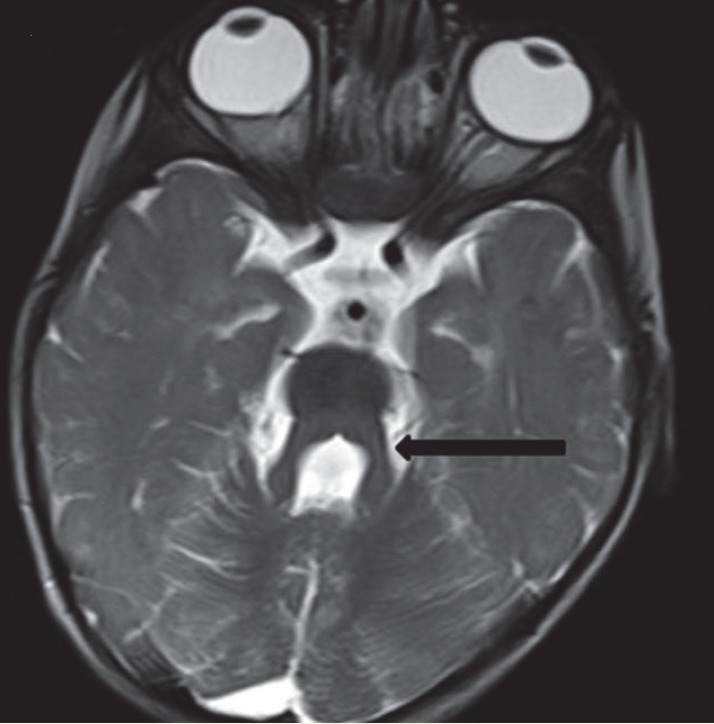
Thickened and elongated superior cerebellar peduncles (arrow) with deepened interpeduncular fossa giving a batwing appearance of fourth ventricle in Joubert’s Syndrome in a 1 year old child

**Fig 3:**
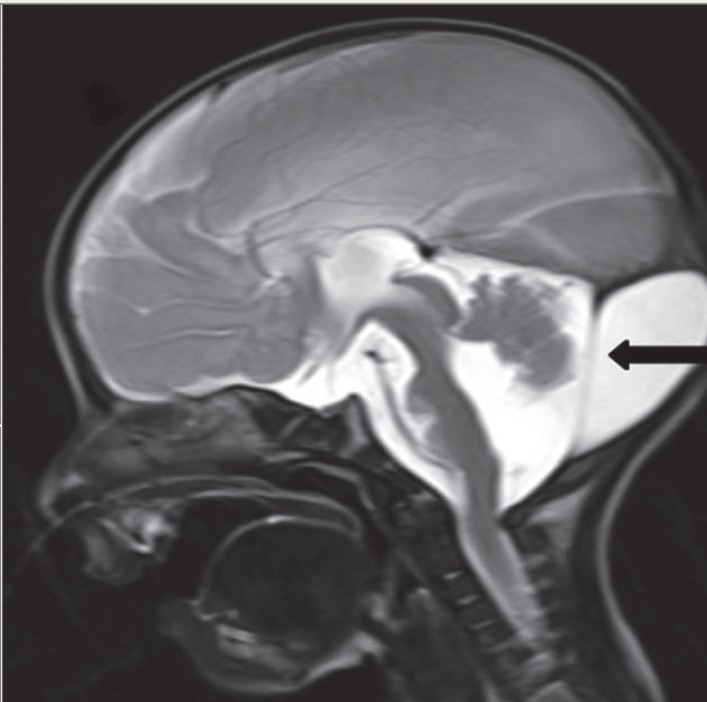
sagittal T2 W sequence shows a dysplastic cerebellum with an enlarged posterior fossa.(black arrow).

**Fig 4:**
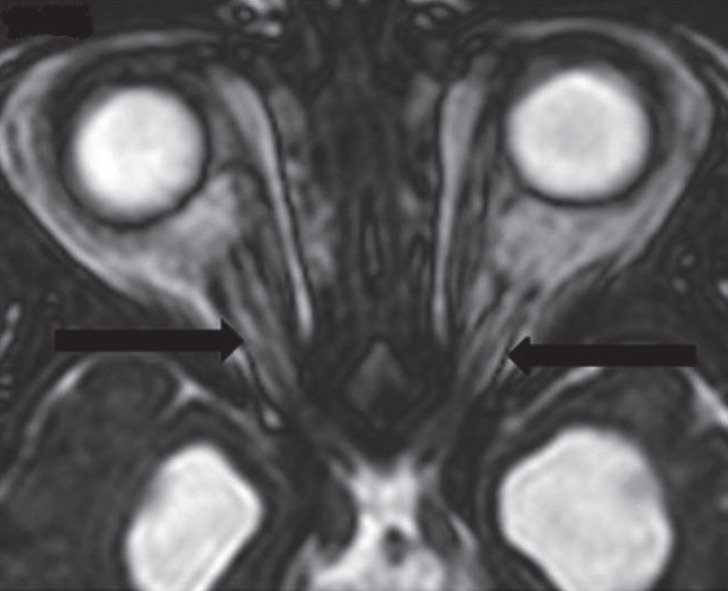
Aaxial T2 W SSFP image shows bilateral thin hypoplastic optic nerves (arrows

**Fig 5:**
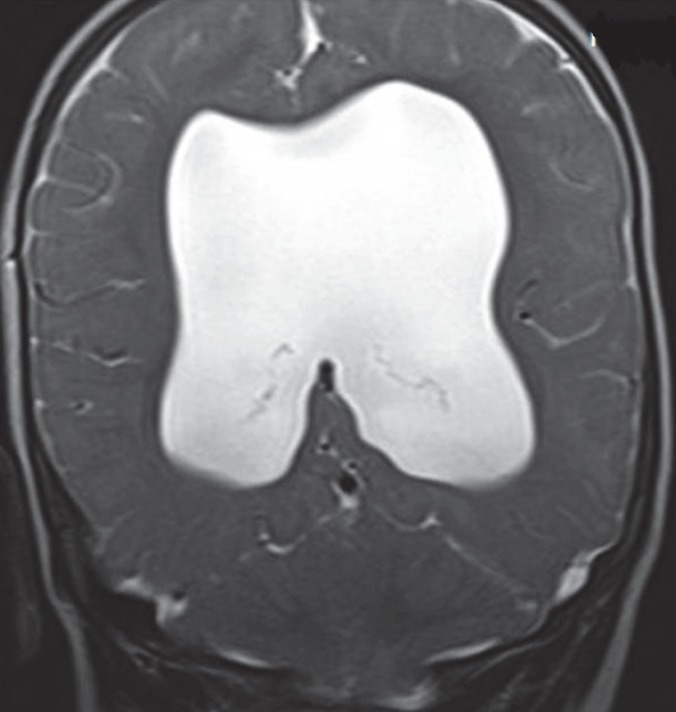
coronal T2 W image shows absence of septum pellucidum with ventriculomegaly in Septo-Optic dyplasia in a 2-year-old male child

**Fig 6:**
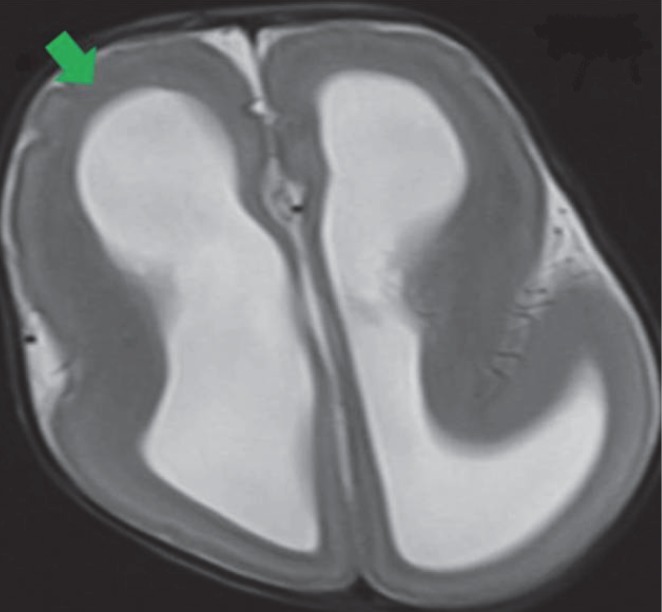
MRI axial T2 weighted images show reduction in normal sulcation smooth agyric cortex (green arrow) with hydrocephalus in type I Lissencephaly

**Fig 7:**
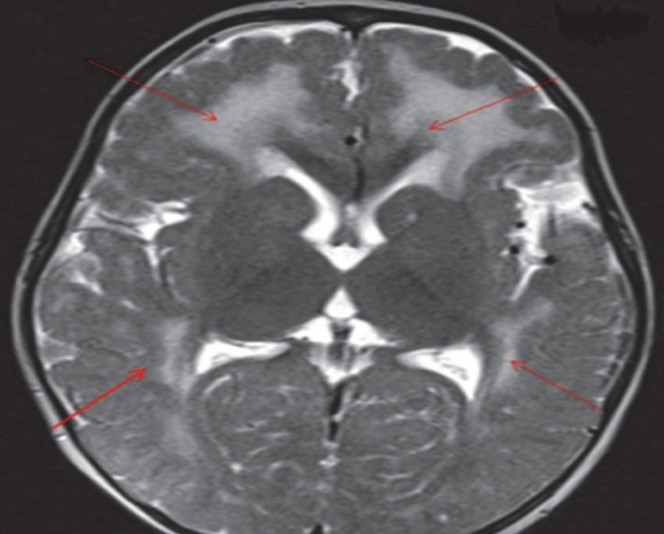
T2W Axial image shows symmetrical white matter hyperintensities (red arrows) in bilateral cerebral hemispheres with thickened pachygyriccortex

Variable degree of posterior fossa abnormalities were observed in the present cases: Jouberts Syndrome, Chiari I, WalkerWarbug Syndrome (WWS) and Fukuyama. Small posterior fossa and tonsillar herniation was seen in Chiari I malformation (Figure 1), Vermian Agenesis, thickening and elongation of the superior cerebellar peduncles and hypoplastic pons in Jouberts syndrome (Figure 2) and a large posterior fossa in WWS (Figure 3). Pathognomic findings on MRI, which enabled a quick diagnosis of Joubert Syndrome, are molar tooth sign due to stretched cerebellar peduncles (black arrow in Figure 2) considered as hallmarks of the anomaly. A large posterior fossa with posteriorly communicating fourth ventricle and elevated torcula was seen in WWS (black arrow in Figure 3) while a small posterior fossa with stenosed foramen magnum( curved black arrow in Figure 1)attributed for the holocord syrinx seen in our case of Chiari I malformation (straight black arrow in Figure 1). Optic nerve and corpus callosal hypoplasia were associated findings seen in septooptic dysplasia (Figure 4, 5). Thickened pachygyric cortex with paucity of sulcation was seen in WWS and in Fukuyama disease (green arrow in Figure 6, red arrows in Figure 7). Associated hydrocephalus was a common feature in most of these cases owing to small foramen magnum and CSF flow obstruction in Chiari I malformation and brain substance loss in Fukuyama and metabolic encephalopathies.

**MRI findings in metabolic disorders**

**Fig 8:**
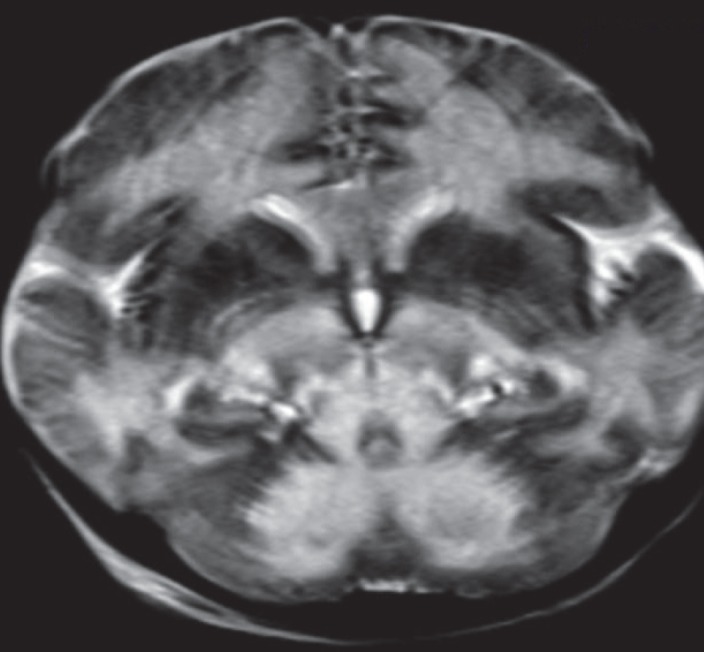
T2W Axial image shows symmetrical white matter hyperintensities in bilateral cerebral hemispheres, thalami, brain stem and deep white matter of cerebellar hemisphere

**Fig 9:**
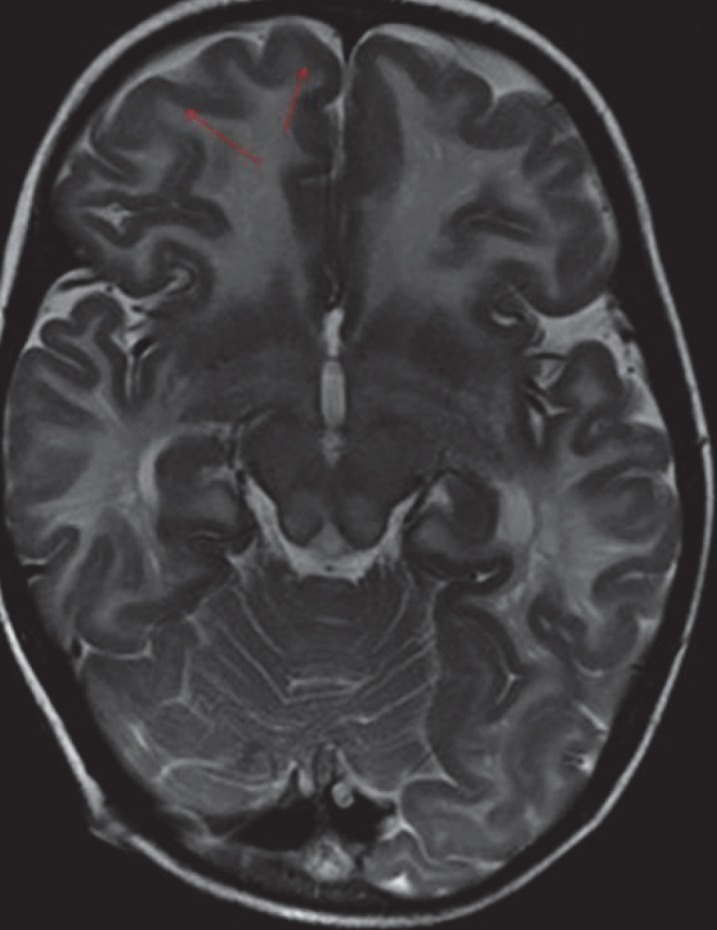
T2W axial image in this child with canavans disease showed diffuse symmetrical hyperintensity in white matter including subcortical U fibers (red arrows), corticospinal tracts, cerebellar peduncles

**Fig 10:**
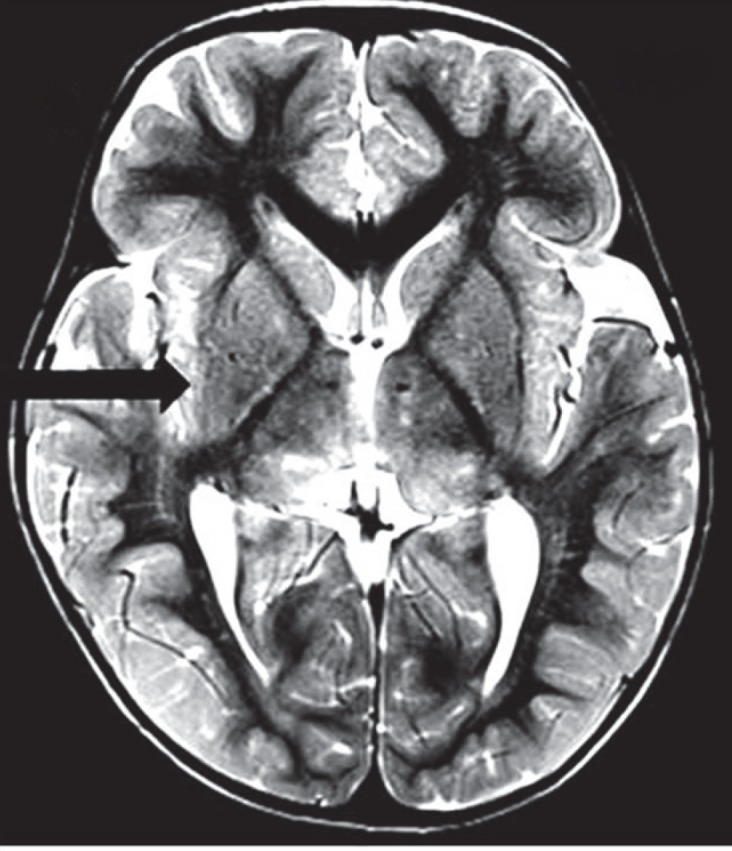
T2W axial image in a case of Leigh’s disease in a 3-year-old child shows hyperintensities in the right globus pallidum and putamen (arrow), bilateral thalami

**Fig 11:**
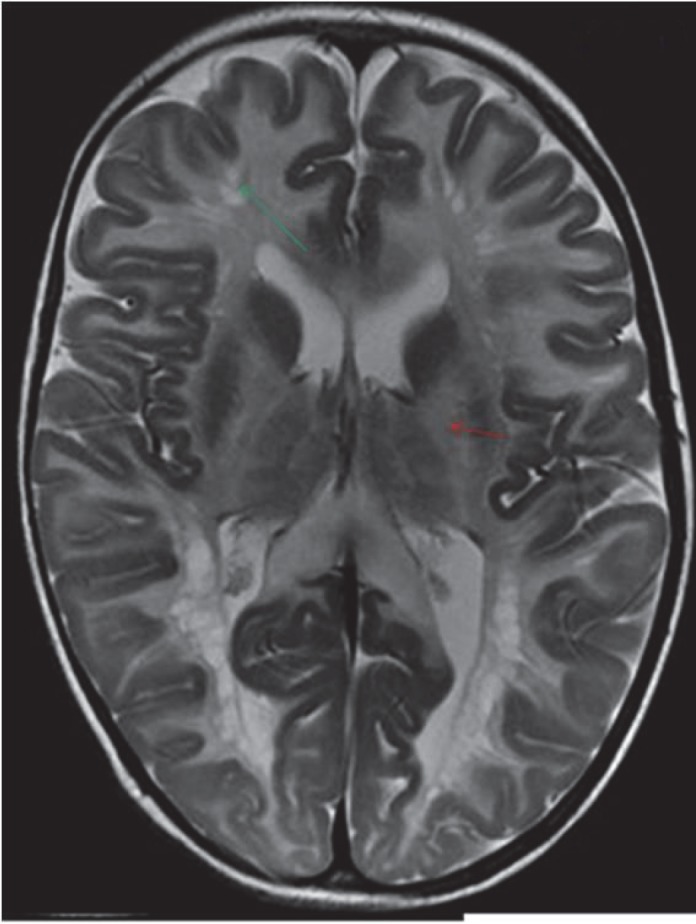
T2W axial image shows cystic degeneration in deep white matter (green arrows) and involvement of internal capsules (red arrows

**Fig 12:**
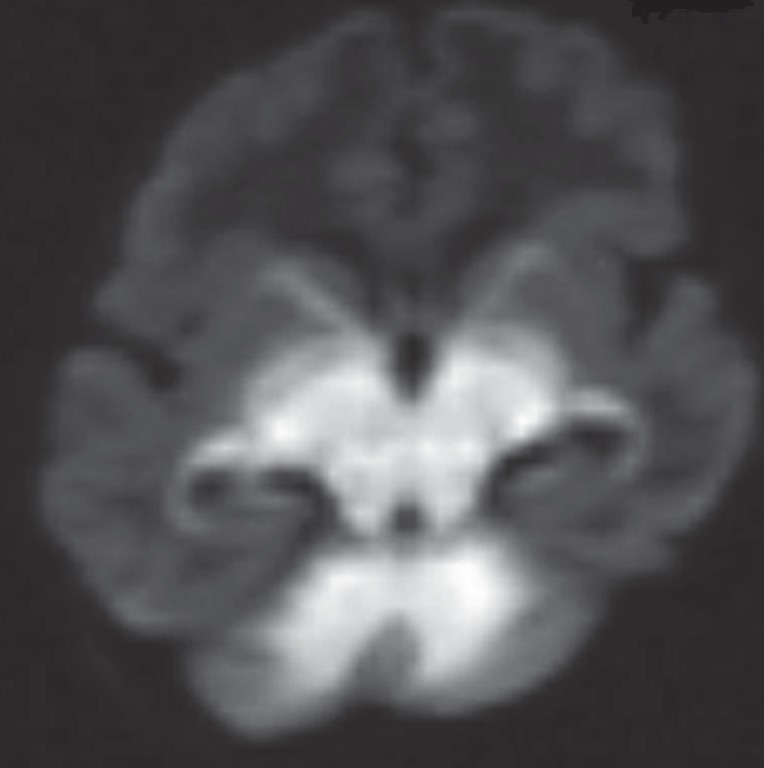
DWI shows hyperintensity in the myelinated white matter areas

**Fig 13:**
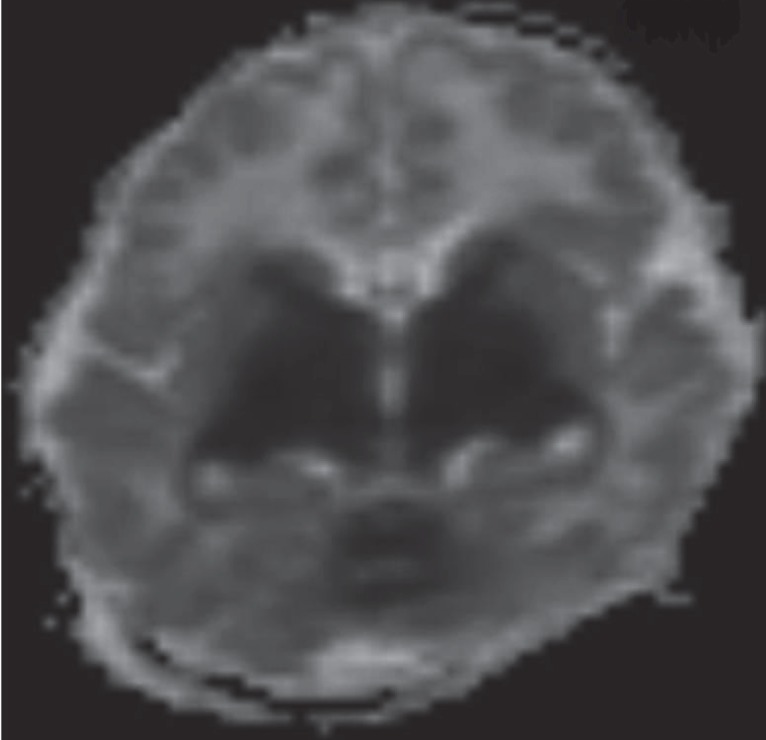
Corresponding low ADC values seen in these DWI bright areas indicative of cytotoxic oedema in this child with Maple syrup urine disease

**Fig 14:**
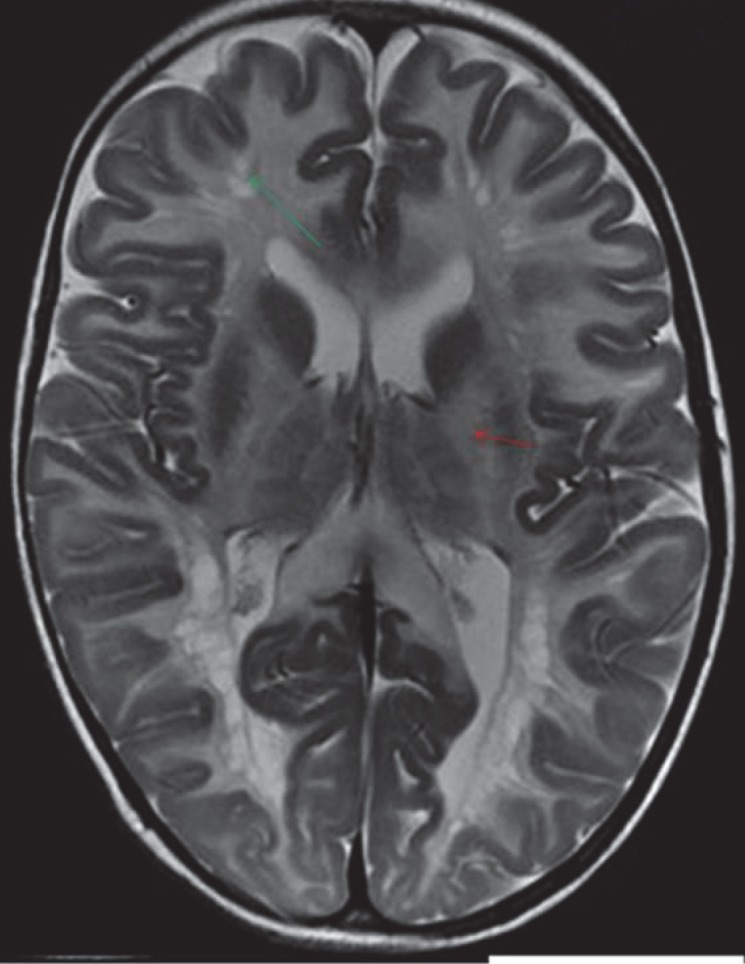
T2W axial image shows cystic degeneration in deep white matter (green arrows) and involvement of internal capsules (red arrows

White matter signal abnormalities were observed in most of the cases with metabolic disorders with symmetrical involvement being the most common type. While the white matter signal changes were confined to the white matter of cerebral hemispheres, corona radiata in adrenoleukodystrohy and metachromatic leukodystrohy diffuse signal changes with involvement of posterior fossa was seen in Canavan’s disease and in Maple syrup urine disease (Figure 8, 9). Among our cases, Basal ganglia involvement was seen in Maple Syrup Urine Disease (MSUD), in Leigh’s disease (Figure 10) and in Canavans disease (Figure 11). MRI shows diffuse swelling of the brain due to extensive edema of the white matter in MSUD. Axial diffusionweighted MR images show areas of hyperintensity restricted on apparent diffusion coefficient maps in posterior limbs of internal capsule (3). (Figure 12, 13). In Canavans disease signal abnormalities were seen in the globuspallidus and thalamus, with sparing of the caudate and putamen (red arrow in Fig 14) similar to earlier literature (4). In all other diseases, no diffusion abnormalities were noted. Sub cortical U fibre involvement was seen in Canavan’s disease (Figure 9) however sparing of arcuate fibers and a rim of uninvolved subcortical white matter was seen in Metachromatic Leukodystrophy, though these differences become obscure in advanced stage of the disease (4). Prominence of the extra cerebral CSF spaces, cisterns and ventricles was seen in most of these cases due to brain substance loss. The MRI pictures in advanced cases of inborn metabolic errors is quite overlapping with diffuse signal abnormalities and volume loss, hence an early scan can help in distinguishing the subtle difference in imaging patterns of individual cases. MR Spectroscopy showed an elevated NAA (N-Acetyl Aspartic Acid) peak in Canavans disease, lactate peak in Leigh’s disease and a small peak at 0.9-1 ppm in MSUD which accounted for the branched chain amino acid metabolites.

**MRI findings in combined hypotonia**

**Fig 15:**
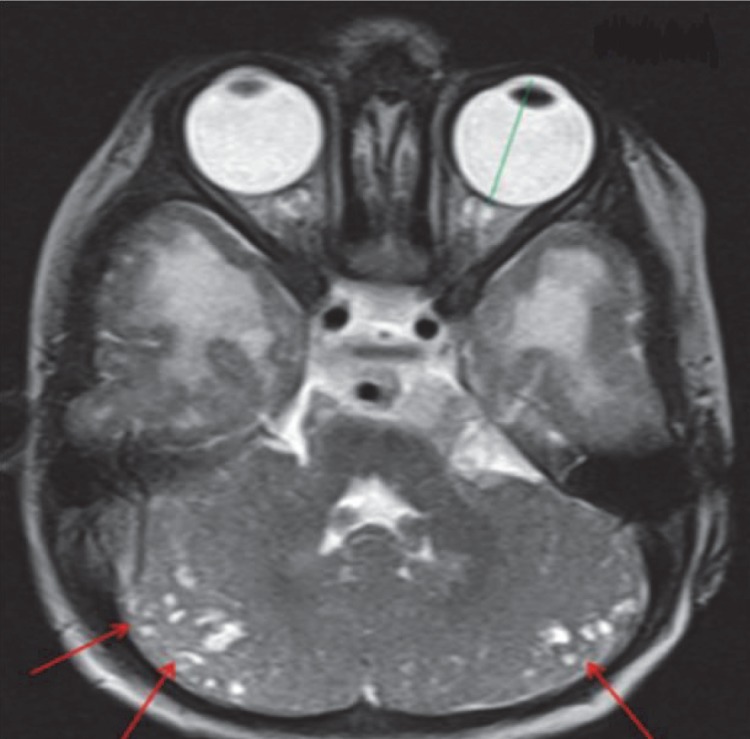
A-T2W Axial image shows multiple small subcortical cysts in bilateral cerebellar hemispheres (red arrows) with enlarged axial length of the globe (green line) in this child with combined hypotonia (Fukuyama disease

In Fukuyama disease, MRI revealed multiple subcortical small cysts in bilateral cerebellar hemisphere and polymicrogyric cortex with diffuse signal changes in white matter of brain (Figure 15). In Walker Warburg Syndrome MRI revealed a thickened featureless cortex with gross paucity of gyri and sulci dilated ventricles and hypoplastic corpus callosum (Figure 3,Figure 6). Though visual abnormalities (a common feature in entities of Congenital Myotonic Dystrophy group) was clinically reported in both of our cases we did not observe any gross ocular abnormalities on MRI, except a mildly enlarged axial length of the globes in Fukuyama (Figure 15) where the child had myopia. Along with structural abnormalities, white matter changes were observed only in Fukuyama disease (Figure 7). MRI findings combined with raised creatine kinase and abnormal EMG pattern enabled diagnosis of these two cases.

**Table 1:** Clinical evaluation for localization of hypotonia

	**AGE/SEX**	**Family/antenatal/perinatal history**	**Clinical presentation/s**	**Dysmorphic facies or other significant physical malformations**	**Tendon reflexes**	**Muscle mass and sensations in extremities**	**Associated psychomotor /developmental delay and postural /protective responses**	**Biochemical abnormalities/Specific tests**
1.MSUD	10 days/M	Consanguineous marriage, previous sibling died undiagnosed at the age of 6 weeks, Uncomplicated normal delivery with normal APGAR score at birth	Poor feeding, failure to thrive , depressed level of consciousness ,hypotonia,2 episodes of seizures	-	Reduced	N	+ , Loss of postural /protective responses	Urine gas chromatography detecting high Concentrations of branched keto acids in urine and high levels of BCAA in plasma
2.Canavans	10 months/F	Nil	Poor head control, macrocephaly, generalized hypotonia, poor response to visual stimuli	-	N	N	+, Loss of postural /protective responses	MR spectroscopy revealed elevated NAA peak. High concentration of NAA in urine, plasma
3. MLD	2 years/M	No significant history	Progressive loss of milestones, hypotonia and unstable gait for 3 months	-	N	N	++	EEG revealed diffuse slow waves, Blood leukocyte enzyme assay showed arylsulfatase deficiency
4.X- linked ALD	8 years/M	Sibling brother death at the age of 7 years	Slow, unsteady gait, dysarthric speech , hearing impairment , lethargy and poor finger coordination. Initial hypotonia replaced by gradual spasticity	-	N	N	-	Increased levels of VLCEFA in plasma
5.Fukuyama	9 months/M	No significant history		-	Absent	Reduced++	+	MRI features, Evidence of myotonic dystrophy on EMG, muscle biopsy
6.Joubert Syndrome	1YO/M	-	Hypotonia, delayed milestones	_	N	N	Ataxia, occulomotor, apraxia	MRI -Molar tooth sign and vermian hypoplasia
7. SOD	2/m	nil	Poor motor incoordination and hypotonia, failure to thrive	Dolicocephaly and strabismus	N	nil	++	MRI-Absent septum pellucidum and hypoplastic otpic nerves, decreased groth hormone levels , increased optic disc to mascular diameter ratio.
8. Walker Warburg syndrome	2m/m	Consanguinois marriage , full term, Caesarean section	Hypotonia, seizuresand ftt and microphthalmia	Macrocephaly	Decrease	N	Loss of postural and protective responses	MRI-lissencephaly pachygyria, hydrocephalus, cerebellar hypoplasia
9. Leigh syndrome	3Y/F	NIL	Hypotonia, myopia	Mypoia, strabismus	N	N	Mild motor weakness	Elevated serum and CSF lactate level
10.Chiari I with holochord syrinx	8/m	nil	Hypotonia,headache, muscle weakness	Myopia, strabismus, ataxia	N	Mild atrophy	+	MRI -Cerebellar tonsillar herniation with holochord syrinx

**Table 2:** MRI Brain findings

	Brainstem and posterior fossa involvement	White matter edema-severity pattern	Loss of brain substance	Basal ganglia involvement	Any structural malformation (eg.corpus callosal agenesis)	Abnormality on DW MR and MR spectroscopy
1.MSUD	+	++,diffuse symmetrical	+	+	-	Diffusion restriction in areas of myelinated white matter, peak at 0.9-1 ppm on MRS
2.CANAVANS	+	+++	++	++	-	Elevated NAA peak
3.MLD	-	++	-	++	-	-
4.X Linked ALD	-	++	++	-	-	Trilaminar edge enhancement on contrast study
5.Fukuyama	Cerebellar subcortical cysts	++	-	-	+	nil
6. Jouberts Syndrome	Molar tooth sign, vermian hypoplasia with elongated and thickened superior cerebellar peduncle and bat wing sign	_	_	_	Vermian hypoplasia with elongated and thickened superior cerebellar peduncle	_
7.Septo-optic Dysplasia	_	_	+	_	Septum Pellucidum complete absence, optic nerve hypoplasia, thinned out corpus Callosum	_
8.Walker Warburg Syndrome	Cerebellar vermian hypoplasia with enlargement of posterior foassa	+	+	_	Lissencephaly, pachygyria,	Nil
9. Leigh disease	++	_	_	+	_	Elevated choline on MRS
10. Chiari I with holochord syrinx	Tonsillar herniation of 10mm below the foramen magnum with compression of retrocerebellar CSF space	_	_	_	_	_

## Discussion

Advances in neuroimaging have led to early recognition and diagnosis of rare congenital anomalies of brain and inborn metabolic disorders in floppy infants even before the full clinical manifestations of the disease sets in the later childhood. Present series was aimed at describing MRI features and its importance in the work up of floppy infants with congenital structural and metabolic basis of neurological disorders. Structural malformations and metabolic encepahalopathies produce profound hypotonia in infants and often in early months of life, this may be the sole manifestation of these rare diseases (5).These infants if left undiagnosed /untreated go on to develop seizures or spasticity in later months of life due to repetitive brain damage. Hence, it is important for the referring pediatrician to have high index of suspicion and consider neuroimaging in these children. After studying MRI features of the brain in present series, three groups were formed as follows: a) Structural Malformations (e.g. Joubert syndrome, septooptic dysplasia) and neuronal migration defects (e.g. Lissencephaly). b) Inborn Metabolic Disorders with altered signal characteristics of white matter and basal ganglia (Leigh’s disease, Canavan’s disease, MLD) c) Essentially normal MRI with no structural or signal changes in children with static disorders. In structural malformations, MRI shows neuronal migration anomalies like featureless thickened cortex with pachy/polymicro gyria, brainstem abnormalities, Callosal agenesis, cleavage abnormalities like fusion of frontal horns in lobar prosencephaly/septooptic dysplasias and ventricular dilatation. Sequential MRI is of great value in assessing the loss of brain substance and predicting the prognosis (7). Metabolic disorders usually affect multiple organ systems and the child can be very sick in addition being hypotonic. Based on MRI features such disorders can be divided into those where the disease process is caused by accumulation of a toxic metabolite due to a defect in enzyme (e.g. MSUD, Canavan’s Disease) and the other group where the child suffers due to defect in energy production (e.g., Mitochondrial disease like Leigh’s disease). White matter signal changes is a constant feature in this group with variable involvement of the basal ganglia and subcortical U fibers help to exclude other entities and formulate close differentials (5, 6). A repeat MRI helps to resolve the problem and the MRI of a developing brain has to be interpreted cautiously, keeping in mind the correct age matched myelination sequence. The Normal Hyperintense Signal on T2W image in the yet to be myelinated white matter of a neonatal brain needs to be differentiated from the abnormal altered signal in leukodystrophies (5, 6). Neuronal migration abnormalities, dysplastic cortex, which can account for the structural causes of central hypotonia often becomes more apparent with growth and on follow up MRI study. The two cases of combined hypotonia included in our case series are Fukuyama disease and Walker Warbug Syndrome where the Tendon Reflexes were lost with an elevation of creatine kinase as well as visual disturbances and delayed developmental mile stones thus including features of central as well as peripheral hypotonia. These typical MRI features (e.g., Multiple sub cortical small cysts in bilateral cerebellar hemisphere and polymicrogyric cortex with diffuse signal changes in white matter of brain) help to diagnose this disease from a complex heterogenous group of disorders commonly termed congenital muscular dystrophies. Muscle biopsies in these cases of combined hypotonia will not give any clue to the specific etiology excepting muscular dystrophy. Hence, the MRI is a rational investigation in this group of combined hypotonia as well (7, 8). Symmetrical signal abnormalities of the basal ganglia (e.g. mitochondrial Cytopathies like in case of Leigh ‘s disease), diffuse white matter involvement with elevated NAA peak in Canavans disease as well as the detection of brain stem and cerebellar abnormalities (e.g., molar tooth sign in Joubert syndrome) are findings that are pathognomonic for specific disorders. MRI Brain findings help in quick diagnosis as well as explaining the prognosis to the concerned family. To maximize the diagnostic yield and avoid other unnecessary investigations in the systematic evaluation algorithm of these children, the referring pediatrician should be in accord with the fellow radiologist and prioritize the need for neuroimaging in Floppy Children after careful clinical assessment. Dawn et al. (2), mentioned MRI brain as the second investigation of choice after a careful clinical examination and increases the diagnostic yield by another 13%. In inconclusive cases, the MRI studies can provide clues in choosing the next level of investigation for further evaluation e.g. test which will establish the particular enzyme disorder/organelle dysfunction etc. Early imaging in patients of Chiari I with hydromyelia has a prognostic value as prompt surgical decompression in the initial stage can reduce morbidity and mortality (9). MRI enables detection of associated abnormalities like optic nerve thinning in septooptic dysplasia, corpus callosal hypoplasia in WWS and septooptic dysplasia, large globes in Fukuyama which helps in explaining the overall clinical picture of these children with global developmental delay. MRI is indeed more accessible and quick investigation to establish such rare diagnoses rather than Genetic studies in specialized laboratories, done to confirm the diseases and as a part of counseling for the parents (2, 5). Thus by classifying MRI findings of the children in our series in three broad groups (as described in results), we intended to find out a systematic imaging algorithm for the practicing radiologist in evaluating a hypotonic child.

**In Conclusion**, Neurological evaluation of a floppy child is an extremely difficult and demanding task and the clinician may be clueless about the next correct investigation to choose among the wide array of highly specialized tests for the diagnosis of central hypotonia. Hence, neuroimaging should be considered for quick diagnosis and identification of potentially treatable cases.

## List of abbreviations:

MSUD=Mapple Syrup Urine Disease, ALD=Adrenoleukodystrophy, MLD=Metachromatic leukodystrophy, SOD=Septo-optic Dysplasia, VLCEFA=very long chain essential fatty acids, NAA=N-Acetyl Aspartic Acid, N-normal, +-(positive /present), - (absent)
